# Investigating the effect of couple-centered counseling by Gottman method on the intimacy of infertile couples referring to the infertility Ward of Fatemieh hospital, Hamadan, Iran in 2020: a quasi-experimental study

**DOI:** 10.1186/s12888-022-04228-z

**Published:** 2022-09-01

**Authors:** Mehrnoosh Hosseinpoor, Seyedeh Zahra Masoumi, Farideh Kazemi, Farzaneh Soltani, Mohammad Ahmadpanah

**Affiliations:** 1 Department of Midwifery and reproductive health, School of Nursing and Midwifery, Hamadan University of Medical Sciences, Hamadan, Iran; 2 Department of Midwifery and reproductive health, Mother and Child Care Research Center, School of Nursing and Midwifery, Hamadan University of Medical Sciences, Hamadan, Iran; 3 Department of Psychiatry and clinical psychology, medicine school, Hamadan University of Medical Sciences, Hamadan, Iran

**Keywords:** Infertility, Marital intimacy, Gottman counseling, Infertile couples

## Abstract

**Background:**

Infertility is among the most unpleasant experiences for couples struggling with it. Therefore, coping with its associated psychological burden has become a concern in many societies due to its negative impacts on couples’ lives and intimacy levels.

Lack of marital intimacy leads to unfortunate consequences such as dissatisfaction with marital relationships between spouses. Therefore, these people should be encouraged to find an effective solution to cope with infertility.

Regarding the importance of the emotional relationship between infertile couples and the success rate of infertility treatment, this study aims to determine the effect of couple-centered counseling by the Gottman method on marital intimacy of infertile couples referred to the infertility ward of Fatemieh Hospital in Hamadan.

**Method:**

The sample of this quasi-experimental study included 60 infertile couples in the evaluation phase of treatment with primary infertility. We collected data in a referral infertility center in Hamadan (Iran) between December 2020 and May 2021. Marital intimacy was measured using Thompson & Walker’s Marital Intimacy Questionnaire. At the beginning of the study, the intervention and control groups completed this questionnaire. The intervention group received Gottman couple-centered counseling based on GATHER principles in 8 sessions. The groups completed the questionnaires 4 weeks after the last intervention session again. If the distribution was normal, the ANCOVA test was used to evaluate the differences between the two groups. Intra-group comparisons were performed using paired t-test, and intergroup comparisons were performed using an independent t-test.

**Results:**

The mean score of female intimacy increased significantly after the intervention (*P* = 0.009). There was no significant difference in socio-demographic characteristics between groups (*P* < 0.05), both of which were adjusted in ANCOVA. After the intervention, the mean marital intimacy scores were significantly higher in women participating in the intervention group (from 75.6 (±10.63) to 78.86 (±7.87)). In addition, after the ANCOVA test, the difference was statistically significant (*P* = 0.009; MD: 3.74, CI: 0.95 and 6.52). The mean score of male marital intimacy increased after the intervention (from 78.93 (±10.21) to 78.9 (±9.79)), although the difference was not statistically significant (*P* = 0.54; MD: -0.58, CI: − 2.51 and 1.34).

**Conclusion:**

The findings support the effective role of Gottman’s couple-centered counseling in increasing marital intimacy by raising couples’ awareness about the principles of proper relationships between them. This outcome suggests that counseling with couples, especially in critical life situations, can improve their relationship in the infertility treatment process and prevent emotional divorce and other negative impacts on their lives.

**Trial registration:**

IRCT Registration Number IR.UMSHA.REC.1399.535, registered on 21/09/2020.

## Background

Infertility is defined as a failure in clinical pregnancy after 12 months of regular and unprotected sex and is classified into primary and secondary categories [1].

The overall infertility rate in the world is 10% [2] and 13.2% in Iran, making it a public health concern worldwide [3].

As a crisis in a couple’s marital life, infertility causes psychological problems and can negatively affect the couple’s adjustment [4] and their sexual and marital relations [5]. In this respect, women may also face problems with their marital intimacy [6].

Numerous studies have reported a significant relationship between marital intimacy and the improved quality of life and couples’ relationship [7].

Intimacy is an interactive dynamic integrative process that includes emotional, psychological, intellectual, and spiritual dimensions [8]. The high level of emotional intimacy in interpersonal relationships is among the strongest predictors of physical and mental health and mutual satisfaction [9]. Couples with a higher level of intimacy may be more capable of coping with problems and changes related to their relationship and consequently experience higher marital satisfaction [10]. Marital intimacy is a basic need in infertile women [11]. In this respect, infertility stress disrupts marital intimacy, lowers self-esteem, and reduces the frequency of sexual intercourse [12]. On the other hand, lack of marital intimacy leads to unpleasant consequences such as relationship dissatisfaction, loss of love and affection, and poor understanding in relationships between spouses [13].

Nowadays, there are various ways to increase the quality of marital life and train marital communication skills [14], one of which is counseling. Infertility counseling aims to search, understand, and solve the problems caused by infertility and its related treatment and find ways to deal with it effectively. The counseling process should consider the needs of the patient or the client [15].

Various couple therapy and family therapy approaches have been developed to minimize conflicts and communication disorders between couples and help them better deal with current problems [16]. One of these couple therapy approaches is Gottman’s counseling. Gottman’s seven principles are based on love and affection between couples to help rebuild the relationship between couples. Overall, this method is used to increase the intimacy of all couples [17].

Gottman’s method is based on observation of couple therapy, trying to help couples achieve a deep understanding of cognition, awareness, and empathy [18] and change their thoughts, perceptions, and behaviors [19]. Gottman couple therapy is primarily aimed at helping couples rebuild and strengthen friendly relationships with each other [17] and ultimately leads to their proper intimacy and interpersonal growth [18]. It also helps couples learn the differences between them and understand that they will remain forever; accordingly, they must learn to deal with them [20]. Gottman et al. found that positive affection was the best predictor of relationship stability over a 6-month period among newlyweds and, conversely, non-emotional conflict was associated with communication dissatisfaction [21]. In Gottman’s training program, teaching effective communication skills can help couples establish an efficient two-way relationship for growth and prosperity, reduce the persistence of destructive and negative feelings during marital relationships, and bring a relationship with greater intimacy and satisfaction for couples [22].

Midwives play an important role in training and counseling at various stages of women’s lives and, consequently, in the life of couples. Therefore, the training and counseling they provide to infertile couples play an important and undeniable role in the lives of these couples. The high prevalence of infertility and its negative impacts on couples’ marital intimacy provides a golden opportunity for interventions and counseling.

Soheili et al. (2020) concluded that group counseling based on Gottman couple therapy has been effective in the marital intimacy of female nurses [22, 23]. Also, Saemi et al. (2019), using the Gottman couple therapy method and emotion-based couple therapy, showed the efficiency of marital intimacy in couples’ relationships [24].

Infertility prevention is almost a neglected issue in health systems [25], and only few countries provide legal authorization for infertility counseling and supportive care in their reproductive health policies and programs [26]. To our knowledge, the Gottman method has received limited attention in infertile couples and infertility centers of the studied country. Therefore, the present study uses this method to increase the marital intimacy of infertile couples.

## Method

### Research design and participants

This research is a quasi-experimental study in two groups to investigate the effect of Gottman’s couple-centered counseling on marital intimacy in infertile couples visiting the infertility ward of Fatemiyeh Hospital, Hamadan. The study samples included 60 couples with primary infertility and one treatment failure. The inclusion criteria for couples were primary infertility with one treatment failure experience, the age range of 18–40 years, known as an infertile couple since the last 1 to 5 years, no mental illness, sexual dysfunction, or an underlying disease affecting fertility, and not receiving marital intimacy counseling in the past. Exclusion criteria were: having a history of divorce, severe marital conflict requiring counseling, stress due to serious illness or death of first-degree relatives, and pregnancy for women.

### Sampling and randomization

This research was approved by the Ethics Committee of Hamadan University of Medical Sciences under the codes of IR.UMSHA.REC.1399.535 and IRCT IRCT20120215009014N368 and project NO 9909046166. All participants signed a research consent form.

Sampling was initiated after obtaining the ethical code from the ethics committee of Hamadan University of Medical Sciences. By attending the infertility ward of Fatemieh Teaching Hospital in Hamadan, the researcher extracted a list of infertile couples with one treatment failure. Then, objectives were called and introduced with the research method.

Initially, 100 couples were screened, of which 60 couples were selected. Not meeting the criteria for inclusion, immigration, and secondary infertility were the reasons for eliminating some couples.

The eligibility of the samples was assessed, and eligible couples who wished to participate in the study were invited. After obtaining informed consent from the participants, they were selected by convenience sampling. Next, they were divided into control and intervention groups based on a predetermined allocation sequence. The allocation sequence was determined using blocks with a block size of 4, and the sequence was determined before the intervention. Accordingly, the type of intervention was written in closed opaque envelopes and coded in the order of their sequence.

**Fig. 1:**
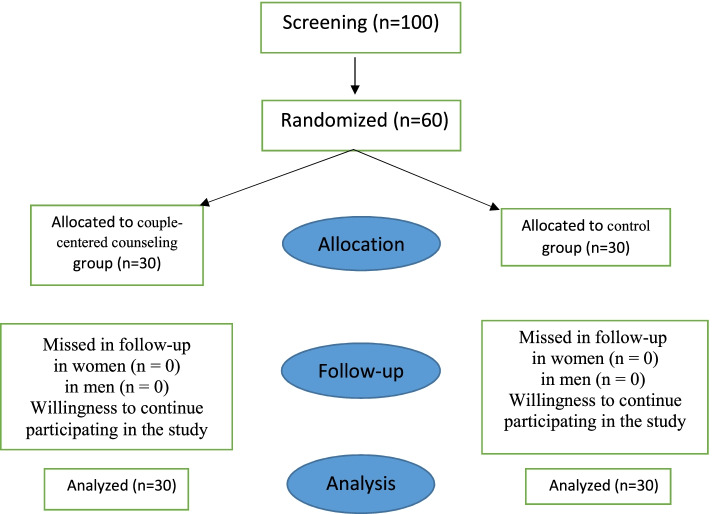
Flowchart Selection of participants in the study. Members of both experimental and control groups completed the Walker and Thompson’s (1983) Marital Intimacy Questionnaire

Each participant was given an envelope after entering the study and asked to complete a demographic information questionnaire. Thus, they were assigned to two groups using the block randomization method (Fig. 1).

### Intervention

In addition to the usual infertility procedures (including medical care and nutrition counseling, ultrasound, and tests at regular intervals), the intervention group received face-to-face couple-centered counseling based on the Gottman method and GATHER (Greet, Ask, Tell, Help, Explain, and Return) principles. These procedures were eight 45-to-60-minute sessions with an interval of at least 1 week conducted at the IVF center of Fatemiyeh Hospital.

In addition to face-to-face sessions, the clients were provided with a counseling package, including Gottman counseling CDs and exercises related to each session. Also, the researcher provided couple-centered online counseling in cases of couples’ requests. On the other hand, the control group received only the routine care provided in the infertility ward. After the counseling sessions and 4 weeks after the last session, both questionnaires were completed again by the experimental and control groups, and the Gottman counseling package was provided to the control group. The training content of the counseling sessions is presented in Table 1.

**Table 1:** Educational content of Gottman counseling sessions based on GATHER principles

Session	period of time	Content	Compliance with GATHER principles
1st session	45–60 min	Welcoming and communicating, introducing and introducing the counselor and clients to each other, explaining the details and goals of counseling sessions, asking open-ended questions in order to get information from couples and actively listening to their answers, giving an introduction to the **Gottman method** (7 principles Gottman), Familiarity with the theory of the **sound relationship house**, Familiarity with the unfortunate beginning and the 4 riders of destruction (criticism, insult and humiliation, defensive state and the formation of a stone wall), Determining the time of the next meeting	Greet Ask Tell Help Explain Return
2st session	45–60 min	Feedback from the previous session and Examining the problems, familiarizing the participants with the first principle of Gottman (Build love map), giving examples and exercises, determining the time of the next session	Greet Ask Tell Help Explain Return
3st session	45–60 min	Feedback from the previous session, the couple’s familiarity with Gottman’s second principle (Share fondness and admiration), emphasizing the positive impact of marital counseling, paying attention to the marital relationship history of shared memories) and the positive characteristics of the other party, neutralizing the 4 rider destruction with the system Interest and admiration, presentation of practice, scheduling of the next session	Greet Ask Tell Help Explain Return
4st session	45–60 min	Feedback from the previous session, familiarity with Gottman’s third principle (Turn toward instead of away), familiarity with the concept of emotional bank account, teaching soothing conversations for more intimacy, setting the time for the next session	Greet Ask Tell Help Explain Return
5st session	45–60 min	Feedback from the previous session, familiarity with Gottman’s fourth principle (the positive perspective), awkward initiation of conversations and outbursts of negative emotions, emphasis on accepting spouse influence and neutralizing the four riders, trying to correct behavior and express disagreement with respect, providing practice, Determine the time of the next meeting	Greet Ask Tell Help Explain Return
6st session	45–60 min	Feedback from the previous session, familiarity with Gottman’s fifth principle (manage conflict), the way to differentiate solvable problems from permanent problems, for example, the way to start gently in disputes, to determine the time of the next meeting	Greet Ask Tell Help Explain Return
7st session	45–60 min	Feedback from the previous session, familiarity with Gottman’s Sixth Principle (make life dreams come true), overcoming persistent disagreement with logical speech, emphasizing the expression of personal dreams, teaching the acceptance of unresolved disputes by example, setting the time for the next session	Greet Ask Tell Help Explain Return
8st session	45–60 min	Feedback from the previous session, acquaintance with the seventh principle of Gottman (create shared meaning), emphasizing the spiritual part of life and building a common culture, stating the remaining explanations provided and the necessary clarification, if necessary, refer to a psychologist	Greet Ask Tell Help Explain Return

### Data collection tools

Data collection tools included the demographic information questionnaire and Thompson and Walker’s Marital Intimacy Questionnaire. The Social Demographic and Midwifery Questionnaire were prepared by the authors. Ten Hamadan University of Medical Sciences faculty members confirmed this instrument’s validity. Also, the questionnaires were completed by couples separately.

### Demographic information questionnaire

The demographic information questionnaire consisted of 27 items about age, education, duration and type of marriage, infertility duration and type, and life satisfaction.

### Thompson and Walker’s marital intimacy questionnaire

The Marital Intimacy Questionnaire consisted of 17 items with a maximum score of 119 and a minimum score of 17, rated on a 7-point Likert scale from 1 (Never) to 7 (Always). Also, marital intimacy scores were evaluated based on the research objectives.

This questionnaire has been standardized in Iran, as its reliability coefficient was determined to be 0.96 by Nematzadeh (2020) et al. [27].

Reliability was measured by the Intraclass correction (ICC) coefficient. For this purpose, 30 couples completed the Thompson and Walker Marital Intimacy Questionnaire at a retest of 2 weeks. As a result, the ICC was calculated to be 95%, indicating the good reliability of our instrument.

### Ethics approval and consent to participate

This study was based on Gottman’s principles and related guidelines. All participants were included in the study, and their informed written consent was obtained. The ethics committee of Hamadan University of Medical Sciences approved the study (ethical code: IR.UMSHA.REC.1399.535).

### Statistical analysis

Data were analyzed using Stata software ver. 13. Central and dispersion indices were used to describe quantitative variables. Quantitative data were compared between the two groups in terms of demographic and contextual variables by independent t-test, and qualitative data were performed by Fisher test. ANCOVA test was used to evaluate the differences between the two groups in terms of intimacy score and couples’ marital satisfaction due to the normal distribution. In-group comparisons were performed using paired t-test and between groups with independent t-test. The significant level in all statistical tests was considered less than 0.05.

## Results

The mean age of the female participants was 31.87 (± 4.56) and 30.67 (± 4.09) years in the experimental and control groups, respectively, while the mean age of the male participants was 35.47 (± 5.57) and 36 (± 5.25) years in these groups. Also, 43.3% of women had an academic degree, and 50% had a high school diploma in the experimental and control groups. These percentages for men were 40 and 50, respectively. The mean duration of infertility in the experimental and control groups was 5.37 (± 3.38) and 7.97 (± 4.58) years, respectively. Also, the effect of the infertility period on the study results was controlled by the ANCOVA test. About 26.7% of the experimental group reported the cause of infertility as male factors, and 56.7% of the control group reported female and male factors as its cause. In addition, 50% of the experimental group and 56.7% of the control group reported a history of IVF. Participants in both groups were homogeneous in terms of all demographic variables (*p* < 0.05; Table 2).

**Table 2:** Comparison of quantitative and qualitative demographic variables between the two groups

Variable	Gender	Subgroup	intervenion group Mean (standard deviation)	control group Mean (Standard deviation)	statistics	*P*-value
**Age (year)**	female	–	31.87 (4.56)	30.67 (4.09)	−1.07	0.28*
	male	–	35.47 (5.57)	36 (5.25)	0.38	0.7*
**education**	female	Reading and writing	1 (3.3)	1 (3.3)	2	0.7**
		High school	5 (16.7)	6 (20)	11	
		Diploma	11 (36.7)	15 (50)	26	
		University	13 (43.3)	8 (26.7)	21	
	male	Reading and writing	5 (16.7)	0 (0)	1	0.08**
		High school	5 (16.7)	10 (33.3)	15	
		Diploma	12 (40)	15 (50)	27	
		University	12 (40)	5 (16.7)	17	
**Duration of infertility (month)**	–	–	5.37 (3.38)	7.97 (4.58)	2.49	0.01*
**Cause of infertility**	–	Female infertility	3 (10)	7 (23.3)	6	0.65**
		Male infertility	8 (26.7)	5 (16.7)	16	
		Female and male infertility	18 (60)	17 (56.7)	36	
		Unknown cause	1 (3.3)	1 (3.3)	2	
**Type of treatment defeated**	–	IVF	15 (50)	17 (56.7)	30	0.75**
		IUI	12 (40)	9 (30)	24	
		medicine	3 (10)	4 (13.3)	6	

*Independent t-test, ** fisher exact test

The mean score of marital intimacy was 76.20 (13.17) in the control group and 78.86 (7.78) in the intervention group. Although the mean score of the experimental group was higher than the control, the difference was not statistically significant (*P* = 0.34) (Table 3).

**Table 3:** Comparison between intra-group and intra-group marital intimacy of women before and after the intervention

Variable	intervenion group Mean (standard deviation)	control group Mean (Standard deviation)	Mean difference (CI 95%)	statistics	***P***-value*
Before intervention	75.6 (10.63)	75.76 (13.29)	0.16 (−6.05, 6.38)	0.05	0.95
After the intervention	78.86 (7.78)	76.20 (13.17)	−2.66 (−8.27, 2.94)	−0.95	0.34
**Mean difference (CI 95%)**	−3.26 (−5.89, − 0.64)	−0.43 (−1.82, 0.96)			
statistics	−2.54	−0.63			
*P*-value**	0.01	0.53			

*Independent t-test, ** Paired t-test

By adjusting the pre-intervention scores, infertility duration, the mean score of female intimacy after intervention was significantly higher in the experimental group compared to the control group (*P* = 0.009) (Table 4).

**Table 4:** Comparison of marital intimacy scores of women in the control and test groups

Variable	Adjusted mean (sd)*	Mean difference (CI 95%)	F^**^	***p***-value^**^
Intervention group	79.4 (4.96)	3.74 (0.95, 6.52)	7.29	0.009
control group	75.66 (4.96)			

*adjust of scores before intervention, duration of infertility ** ANCOVA test

The results of Table 5 showed that the mean score of marital intimacy in the intervention group was higher than the control group (78.9 (9.79) and 77.7 (9.42), respectively) but this difference was not statistically significant (*P* = 0.63) (Table 5).

**Table 5:** Comparison between intra-group and intra-group marital intimacy of men before and after the intervention

Variable	intervenion group Mean (standard deviation)	control group Mean (Standard deviation)	Mean difference (CI 95%)	statistics	***P***-value*
Before intervention	78.93 (10.21)	77.03 (9.6)	2.55 (−7.02, 3.22)	−0.74	0.46
After the intervention	78.9 (9.79)	77.7 (9.42)	2.48 (−6.16, 3.76)	−0.48	0.63
**Mean difference (CI 95%)**	0.03 (−1.41, 1.47)	−0.66 (− 1.51, 0.17)			
statistics	0.04	−1.61			
*P*-value**	0.96	0.11			

*Independent t-test, ** Paired t-test

By adjusting the pre-intervention scores, infertility duration, the mean score of male intimacy in the experimental group was higher than that in the control group after the intervention, but the difference was not statistically significant (*P* = 0.54) (Table 6).

**Table 6:** Comparison of male marital intimacy scores in control and experimental groups

Variable	Adjusted mean (sd)*	Mean difference (CI 95%)	F^**^	***p***-value^**^
Intervention group	78.59 (3.46)	−0.58 (−2.51, 1.34)	0.37	0.54

*adjust of scores before intervention, duration of infertility ** ANCOVA test

## Discussion

This study aimed to investigate the effect of couple-centered counseling by the Gottman method on the intimacy of infertile couples in Hamadan. The analysis results revealed a statistically significant difference between infertile women participating in the control and intervention groups. In other words, there was a significant difference between women who received training and women who had no training. Also, Gottman couple therapy has been effective in the marital intimacy of infertile women. According to the results of statistical analysis and comparison of the average intimacy scores in men in the post-intervention stage, it is inferred that Gottman-centered couple counseling has some effect on the marital intimacy of infertile men.

Infertility is an important stressor in a couple’s life as it is very difficult to accept failure in pregnancy. In this respect, many couples experience a recurring treatment without progress, and a feeling of weakness in achieving the goal (childbearing) appears in them. Although infertility is not a disease, it can cause significant emotional disturbances. This problem leaves many psychological and social complications and delays and interferes with various aspects of the couple’s performance, including sexual activity, self-confidence, and emotional communication [28]. Experience of painstaking treatments and feelings of rejection, fatigue, confusion, and despair are among the cases that infertile couples face. All of these factors can affect the couple’s emotional connection and, therefore, their success in treatment [29].

Intimacy is an important human need and a dynamic process with internal roots based on mutual trust and respect. In this regard, clinical studies have shown that the root of many marital problems is the lack of intimacy between couples. With the help of his seven principles, John Gottman describes how a marital relationship succeeds or fails. This scholar also offers ways to facilitate change in these relationships through educational, psychological, preventative, and therapeutic interventions. These solutions, called the Seven Principles of Success in Marriage, determine the extent to which couples maintain their friendship, intimacy, and passion [30]. The goal of Gottman couple therapy is primarily to help couples rebuild and strengthen friendships with each other [17] and ultimately lead to proper intimacy and interpersonal growth [18]. In addition, Gottman couple therapy helps couples learn that there are differences between them. The therapy asserts that these differences are not indelible, and couples must learn to adapt to these persistent problems [20].

Gottman considered the following seven principles to achieve the goal: designing a love plan, cultivating love and admiration, turning to each other instead of avoiding each other, accepting the spouse’s influence, solving solvable problems, overcoming permanent problems, and creating common meaning. He also used strategies such as gratitude, early marriage memories, and an emotional bank account to increase intimacy and love between couples [30], which we also relied on in the educational package videos.

The results of this study are consistent with those of Soheili et al. (2020), who showed that group counseling based on Gottman couple therapy has a positive effect on marital intimacy in female nurses [23]. Likewise, Mortazavi et al. (2020) concluded that the relationship prevention and improvement program based on the combined approach of Gottman and Glasser is effective in marital intimacy [28].

The present study’s findings are also consistent with those of Sehat et al. (2021). These researchers reported that combination therapy based on emotion and solution is effective in marital intimacy and marital adjustment and can reduce hostile, reprehensible, and domineering behaviors and communication problems, thereby improving intimacy in conflicting couples [31]. Zarei et al. (2018) examined the effectiveness of group counseling based on choice theory and Gottman’s theory on marital intimacy and marital conflicts in married women. They eventually concluded that group counseling based on Gottman’s theory significantly increases intimacy and reduces conflict [32], which is in line with our findings.

The findings of the present study are opposed to those of Besharat Ghara Maleki (2021), who reported that counseling in the method of forgiveness therapy is ineffective in the marital intimacy of couples with emotional divorce [33]. Also, these results are inconsistent with those of Arabpour (2012), who proposed the Glaser theory is ineffective in improving the intimate relationship of couples [34].

The present study showed that couple-centered counseling as an easy and low-cost method increased marital intimacy among infertile women. In fact, couple-centered counseling is a supportive and solution-based approach that aims to identify problems, suggest appropriate solutions, and ultimately encourage people to change their behavior. This approach covers all components effective in interpersonal relationships. Since couple-centered education is much more effective than educating women alone, many studies have suggested considering the role of education and counseling in future research.

Based on the study results, it can be stated that Gottman places great emphasis on love and respect to deal with the negative aspects of marriage. In the Gottman curriculum, effective communication skills training can help couples establish an effective relationship for growth and prosperity and reduce the persistence of negative and destructive feelings during the marital relationship. Infertile couples may experience more emotional distress and divorce due to the stress of not having a child. However, Gottman’s method helps increase the desire to continue the marital relationship, reduce marital incompatibility and conflict, and provide more intimacy and satisfaction for couples [23].

When women in couple-centered counseling sessions received various training along with related exercises to reach the seven Gottman principles, all of this helped them feel more intimate with their husbands. In counseling sessions, familiarity with conflict resolution and effective communication skills helped couples realize that some problems in their marital relationship may never be resolved, and they need to learn how to deal with and manage conflict. The couple realized that establishing an effective relationship with their spouse could better understand each other’s needs and wants. Accordingly, the degree of intimacy between them would increase. Also, in these sessions, couples were introduced with four incorrect communication patterns called four riders: criticism, insult and humiliation, defensiveness, and the formation of a stone wall. They received explanations about these four factors’ destructive and negative impacts on marital relationships and learned how to replace correct behavior with behavior.

Overall, Gottman’s counseling taught couples, especially infertile women, that they could more easily deal with many of their problems, such as infertility, by creating a common sense, thus increasing marital intimacy among women. Intimate relationships and better communication patterns of these couples can go through the stages of infertility treatment better than before.

### Limitations and strengths

Unlike most interventional studies that focus on training women, the counseling sessions in the present study were based on couples’ relationships and Gottman’s counseling content. Another strength of our work was the preparation of Gottman’s counseling package (counseling videos and practicing the sessions) and its presentation to couples, in addition to face-to-face counseling sessions.

One limitation of our work was the absence of a psychologist in counseling sessions. We resolved this issue by referring the couples to a psychologist upon their request after the last counseling session. Another limitation was the small number of samples due to time constraints, which reduces its generalize ability to the whole community.

Furthermore, the non-cooperation of some men to participate in counseling sessions slowed down the sampling rate. Therefore, we held online counseling sessions for some of them. Besides, some others were excluded from the study due to creating problems in emotional communication.

## Conclusions

The results of this study revealed that Gottman’s counseling is significantly effective in increasing marital intimacy in women and to some extent in men. It is, therefore, recommended to use it in infertility centers to increase marital intimacy because it increases the quality of life and leads to success in infertility treatments. Moreover, training counseling skills such as Gottman’s counseling to midwifery students and healthcare staff, especially midwives working in the infertility ward, will help this group benefit from the positive effect of interventions on mental health and better emotional communication between couples.

## Data Availability

The data supporting the results of this study are available upon request to the corresponding author (Seyedeh Zahra Masoumi, zahramid2001@gmail.com).

## Abbreviations

GATHER: Include 6 steps including greeting and introduction (Greeting), interacting with people and asking open questions about their problems (Ask), giving useful information (Tell), helping people to choose the best solution (Help), encouraging communication improvement Explain topics that are not clear (Explain) and finally follow up and receive feedback and, if necessary, plan for a return visit (Return)
